# A Hitchhiker’s Guide to the BK Galaxy

**DOI:** 10.3389/ti.2024.13873

**Published:** 2024-10-31

**Authors:** Hans H. Hirsch, Camille N. Kotton

**Affiliations:** ^1^ Transplantation and Clinical Virology, Department Biomedicine, University of Basel, Basel, Switzerland; ^2^ Transplant and Immunocompromised Host Infectious Diseases, Infectious Diseases Division, Massachusetts General Hospital, Harvard Medical School, Boston, MA, United States

**Keywords:** kidney transplantation, guidelines, BK virus, polyoma, nephropathy, BK polyomavirus

For the last two decades, patients and transplant clinicians have found themselves being suddenly confronted with the hostile galaxy of BK polyomavirus (BKPyV) while surfing through the kidney transplant universe. Deep thought consultation then revealed the existence of underappreciated worlds full of challenging experiences and poor outcomes as well as daring suggestions on how to rescue the journey and to reduce short- and longer-term damage. This seemingly endless odyssey has been accompanied by an expanding and contracting information space, occasionally brightened by short-lived shooting stars, most of them with limited impact for down-to-earth practice. What is more, the mere existence of the BK galaxy, its focal impact and dire costs eventually needed to be communicated to the key passengers of this journey, patients and their relatives, most of whom had possibly never heard of this nebulous conglomeration before. Two years ago, however, a brave mission was concluded by 55 people who had accepted the challenging invitation by The Transplantation Society (TTS) to embark on six working groups with the task to better chart and tackle this not so remote galaxy centering around BK polyomavirus. The *TTS International BK Polyomavirus Consensus Group* safely now returned and published together one of the most updated and comprehensive reports, *The Second International Consensus Guidelines on the Management of BK Polyomavirus in Kidney Transplantation* [1].

Given its significant content and claim, what is a reasonable and lean approach to the BK galaxy, a hitchhiker’s guide facilitating clinical translation and implementation of the new TTS BKPyV guidelines? While the underlying mantra remains regular screening and prompt response to BK polyomavirus-DNAemia by reducing immunosuppression, there are three fixed stars with their own gravity fields, nevertheless clearly interconnected in this travel guide: the *infographic*, the *timeline*, and the *flow-chart*. Rather than being stunned or scared by the collection of tables and their detailed Swiss army knife-like content for every eventuality, we suggest the following approach:

***First*, *consider the infographic***, which is miraculously concise given the encyclopedic character of the updated TTS BK polyomavirus guidelines (Supplementary Figure S1). There, the main recommendations are summarized in their proactive character and directly prepare the quest for more professional and detailed information.

***Second*, *review the conceptual timeline*** after kidney transplantation, which paradigmatically leads through the relevant sequence of virology, immunity and pathology, integrates diagnostic measures and management considerations, and allows for cross-comparison at a given time point (Supplementary Figure S2).

***Third*, *walk through the flowchart*** and explore the suggested decision tree, which gives specific reference to the respective recommendations elaborated in tables of the TTS BK polyomavirus guidelines (Supplementary Figure S3).

These three steps allow to obtain overview, concepts and a first sense of detail - but being primed for now reviewing the current practice in your center is perhaps the most valuable item:

On the positive side, this helps to identify the “*have*” of tools, procedures and staff that are already existing in current center practice as well as those “*must haves*” that are not optimally used or clearly missing.

Given the heavy load and the multidisciplinary character, we presume that the task of harmonizing and successfully translating the updated TTS BK polyomavirus guidelines is best accomplished by a team approach (Figure 1). Perhaps a tabular comparison of “is” *versus* “suggested” or “have” *versus* “must have” provides a first overview and can be complemented by “priorities” and “timelines” to realization in order to create a helpful and traceable planning tool.

**FIGURE 1:**
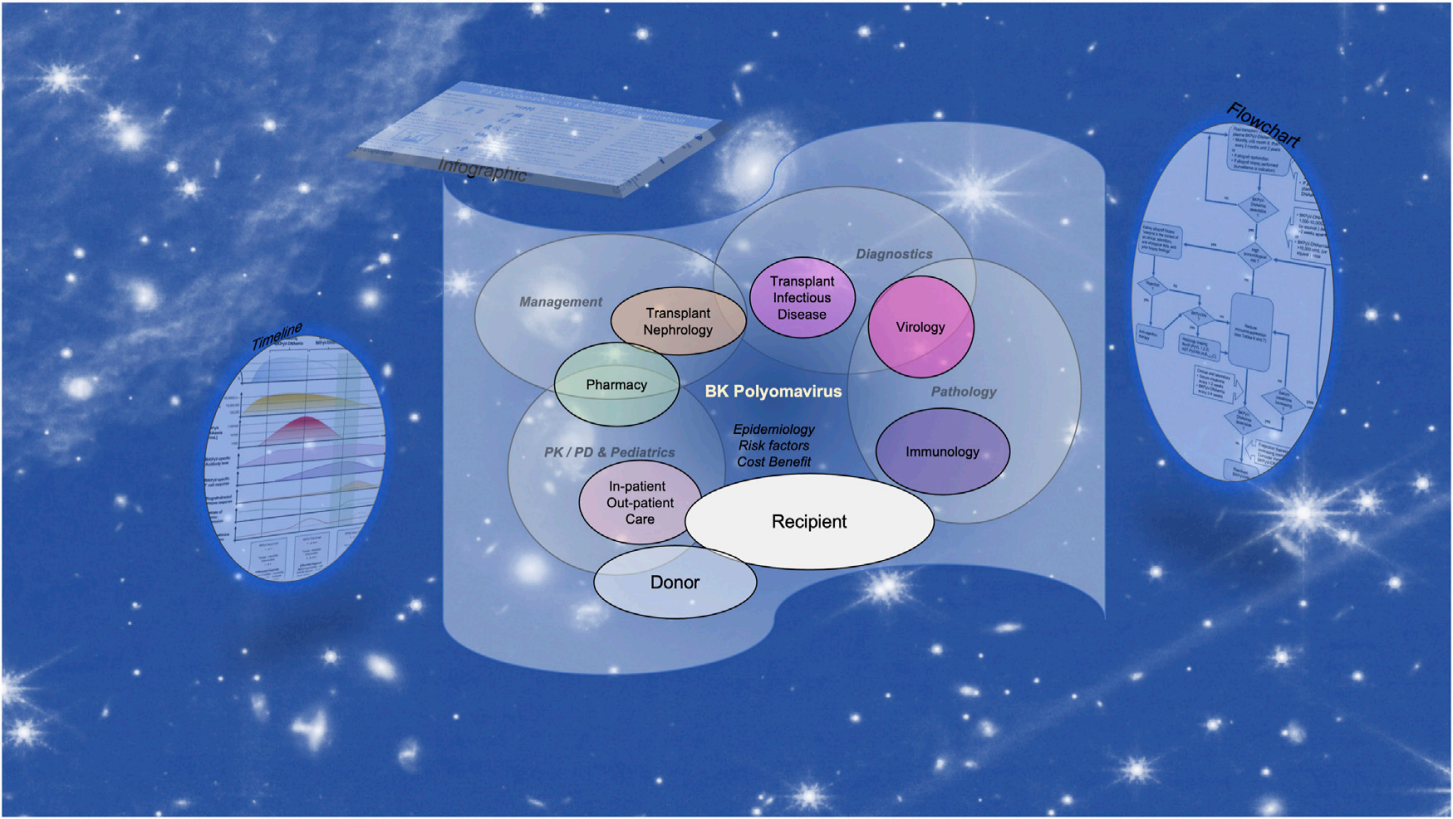
The multidisciplinary challenge of implementing the updated TTS BK polyomavirus guidelines for developing and updating standard operating procedures.

One of the key deliverables is the timely reduction of maintenance immunosuppression guided by a significant plasma BK polyomavirus DNA load. For standard immunological risk and patients with baseline renal function, there is no biopsy required. There is currently no data from randomized clinical trials supporting superiority of either management *strategy 1* (first step reducing mycophenolate) or of *strategy 2* (first reducing tacrolimus). Though frequently mentioned or considered for other reasons, we like to emphasize that there currently is, for no other opportunistic complication posttransplant, more consistent and better documented evidence of feasibility, for rates of success or harm than for the deliberate reduction of immunosuppression for BK polyomavirus. Clearly, trigger and timing remain key determinants [2].

To develop the local *standard operating procedure*, the active participation of all different experts and providers is expected to not only build and expand a broad foundation of knowledge, but also competence for critical (re-)evaluation. Deviation from the current recommendations always remains an option, but then they are the result of active informed decision instead of ignorance. Broad foundation of knowledge also prepares the transplantation team for participation in randomized clinical trials, which are particularly lacking for management decisions.

The new TTS BK polyomavirus guidelines also identify areas of uncertainty and unmet clinical need, where more excellent research is needed and expected to make a difference for patients on their hopefully timeless journey of kidney transplantation. This starts at transplantation with the investigations addressing the value of donor urine virus loads, donor and recipient BK polyomavirus-specific antibodies, virus-specific cell mediated immunity, biomarkers of allograft damage and differentials of T cell mediated rejection or antibody-mediated rejection, as well as therapeutic, preemptive or prophylactic transfer of humoral and cellular immune effectors. But even for the lower hanging fruits, more conclusive data from randomized clinical trials must be considered valuable. These include evaluation of other tantalizing forces in our management universe such as switching to mTOR inhibitors, perhaps combined with low-dose cyclosporine instead of tacrolimus [3], or for patients with persisting BK polyomavirus-DNAemia on tacrolimus monotherapy to switch to belatacept for maintenance.

Importantly, all of the TTS working group members and their leaders are committed to assist transplant clinicians with their expertise in the management of difficult cases as well as in establishing the best local standard operating procedures. Indeed, the challenges of BK polyomavirus and how it affects a significant part of kidney transplant recipients should be explained to the patients and relatives pre-transplant when preparing for one of the otherwise most successful journeys in modern medicine. As a disclaimer known from others, it remains to conclude that “The *Guide* is definitive. *Reality* is frequently inaccurate” [4].

## Data Availability

The original contributions presented in the study are included in the article/Supplementary Material, further inquiries can be directed to the corresponding author.
