# Supplementary material for: A Hitchhiker’s Guide to the BK Galaxy
Source: Transpl Int. 2024 Oct 31;37:13873. doi: 10.3389/ti.2024.13873 (PMC11560434; doi:10.3389/ti.2024.13873)
Supplement: Supplementary file 1 [file DataSheet1.pdf]

Supplemental Materials

Figure S1: **Infographic**

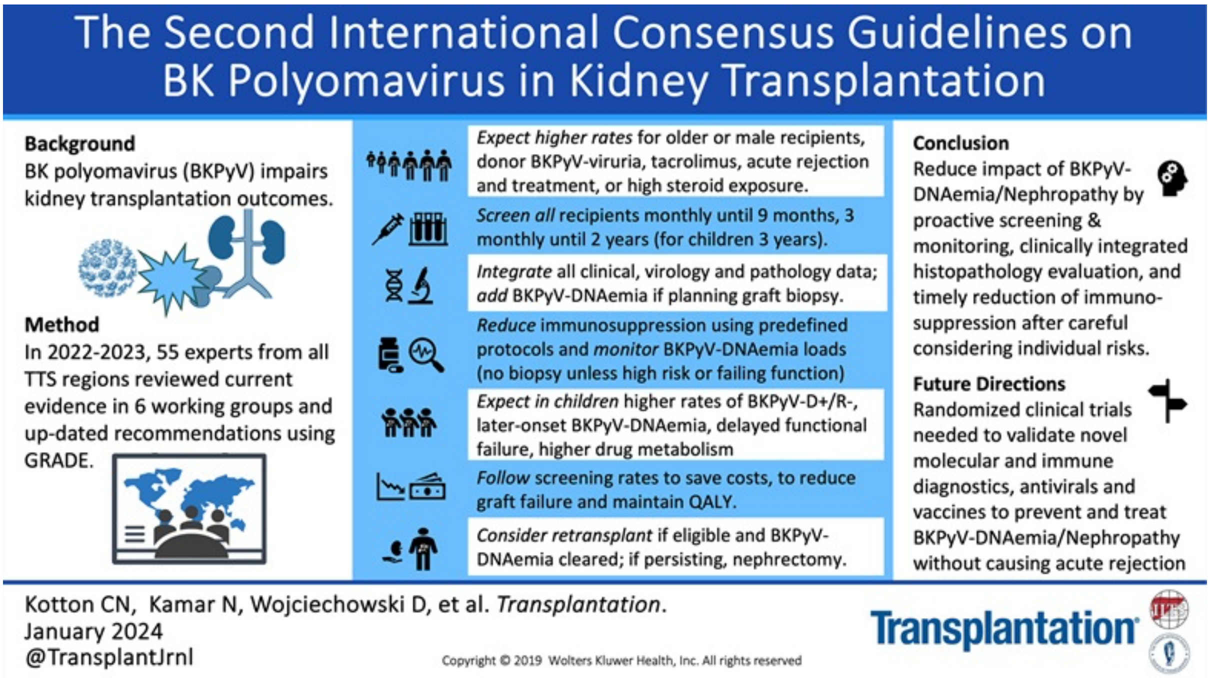

This figure was re-used from:
Kotton CN, Kamar N, Wojciechowski D, et al. on behalf of The Transplantation Society International BK Polyomavirus Consensus Group. The Second International Consensus Guidelines on the Management of BK Polyomavirus in Kidney Transplantation. Transplantation 108(9):p 1834-1866, September 2024. (DOI:10.1097/TP.0000000000004976), and accessed at: [https://journals.lww.com/transplantjournal/fulltext/2024/09000/the_second_international_consensus_guidelines_on.7.aspx].
This figure is published under a CC-BY-NC-ND license and attributed to the original authors.

Figure S2: **Timeline of BKPyV replication and related laboratory and clinical events after kidney transplantation.** (DOI: 10.1097/TP.0000000000004976)
Urinary cytology data mostly describe the onset of decoy cell shedding.
Low-level BKPyV-DNAuria in native urine is detected in <10% before transplantation and increases to high-level BKPyV-viruria defined by decoy cells or BKPyV-DNAuria of >10 million copies/mL of urine.
BKPyV-DNAemia loads are identified in plasma by QNAT approximately 2 to 6 wk after high-level BKPyV-DNAuria.
BKPyV-specific antibody levels increase before immunosuppression is reduced.
As the net state of immunosuppression decreases, rising BKPyV-specific T cell activity is detectable.
The colored dashed lines attempt to capture different scenarios of marker and disease evolution.
Serum creatinine concentration may increase when allograft BKPyV loads and the associated interstitial inflammation become more extensive.
Reducing immunosuppression facilitates antiviral immunity (immune reconstitution), clearance of intragraft replication foci, and clearance of BKPyV-DNAemia loads below the limit of detection while increasing the risk of antidonor immunity and allograft rejection. Increase in serum creatinine may arise because of antiviral immune reconstitution or acute rejection, whereby the former may be transient, unlike the latter.
The shaded green area marks the window of opportunity for reincreasing maintenance immunosuppression to prevent acute T cell–mediated rejection. Potentially accelerated generation of donor-specific antibodies and antibody-mediated rejection are not depicted. BKPyV, BK polyomavirus; c/mL, copies/mL; hpf, high-power field; QNAT, quantitative nucleic acid testing.

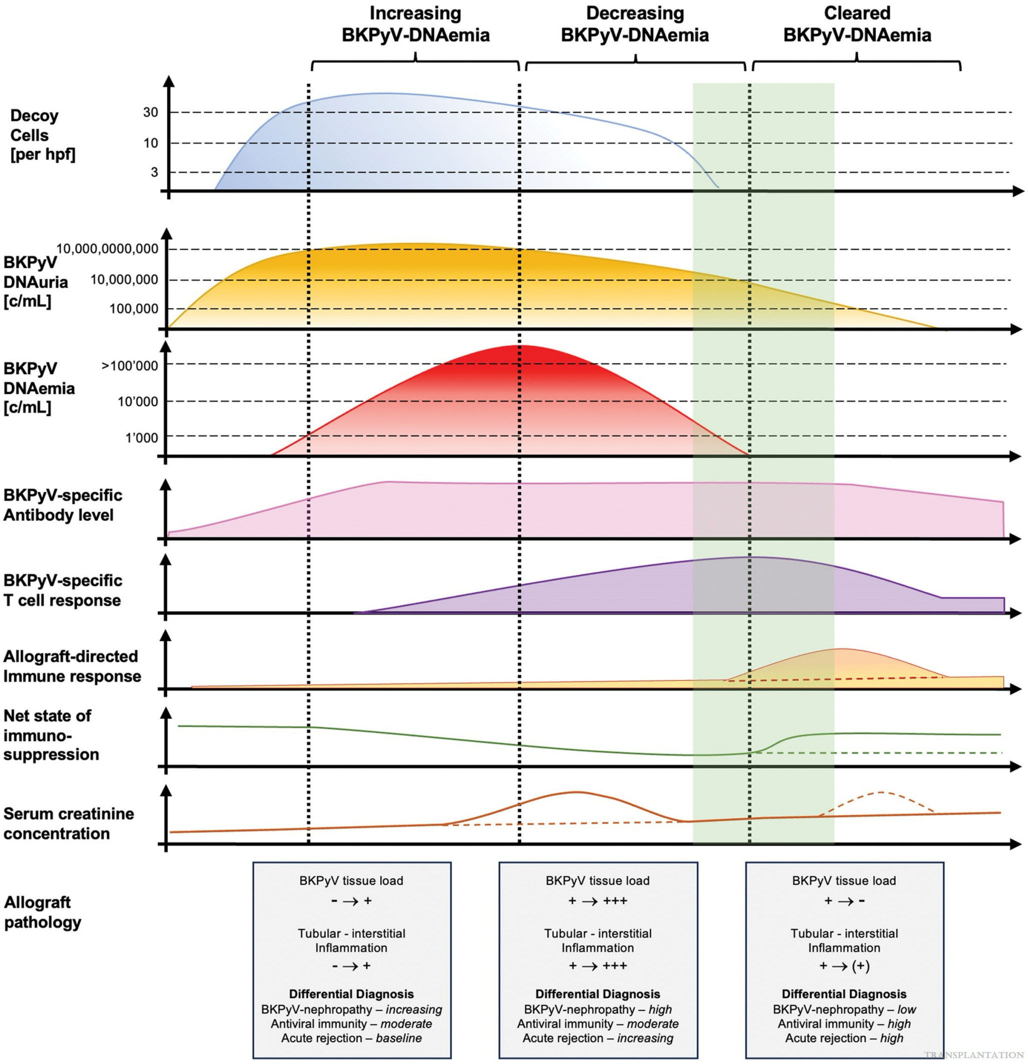

This figure was re-used from:

Kotton CN, Kamar N, Wojciechowski D, et al. on behalf of The Transplantation Society International BK Polyomavirus Consensus Group. The Second International Consensus Guidelines on the Management of BK Polyomavirus in Kidney Transplantation. Transplantation 108(9):p 1834-1866, September 2024. (DOI:10.1097/TP.0000000000004976), and accessed at: [https://journals.lww.com/transplantjournal/fulltext/2024/09000/the_second_international_consensus_guidelines_on.7.aspx].
This figure is published under a CC-BY-NC-ND license and attributed to the original authors.

Figure S3: Flowchart (DOI: 10.1097/TP.0000000000004976)
Integrating screening, diagnosis, and management of BKPyV replication in kidney transplant recipients. For details, see consensus statements and recommendations, including Tables 6 and 7, which describe the principal approaches to reducing immunosuppression. AST, American Society of Transplantation; BK polyomavirus nephropathy; BKPyV, BK polyomavirus; BKPyVAN, PyVL, polyomavirus-tissue load.

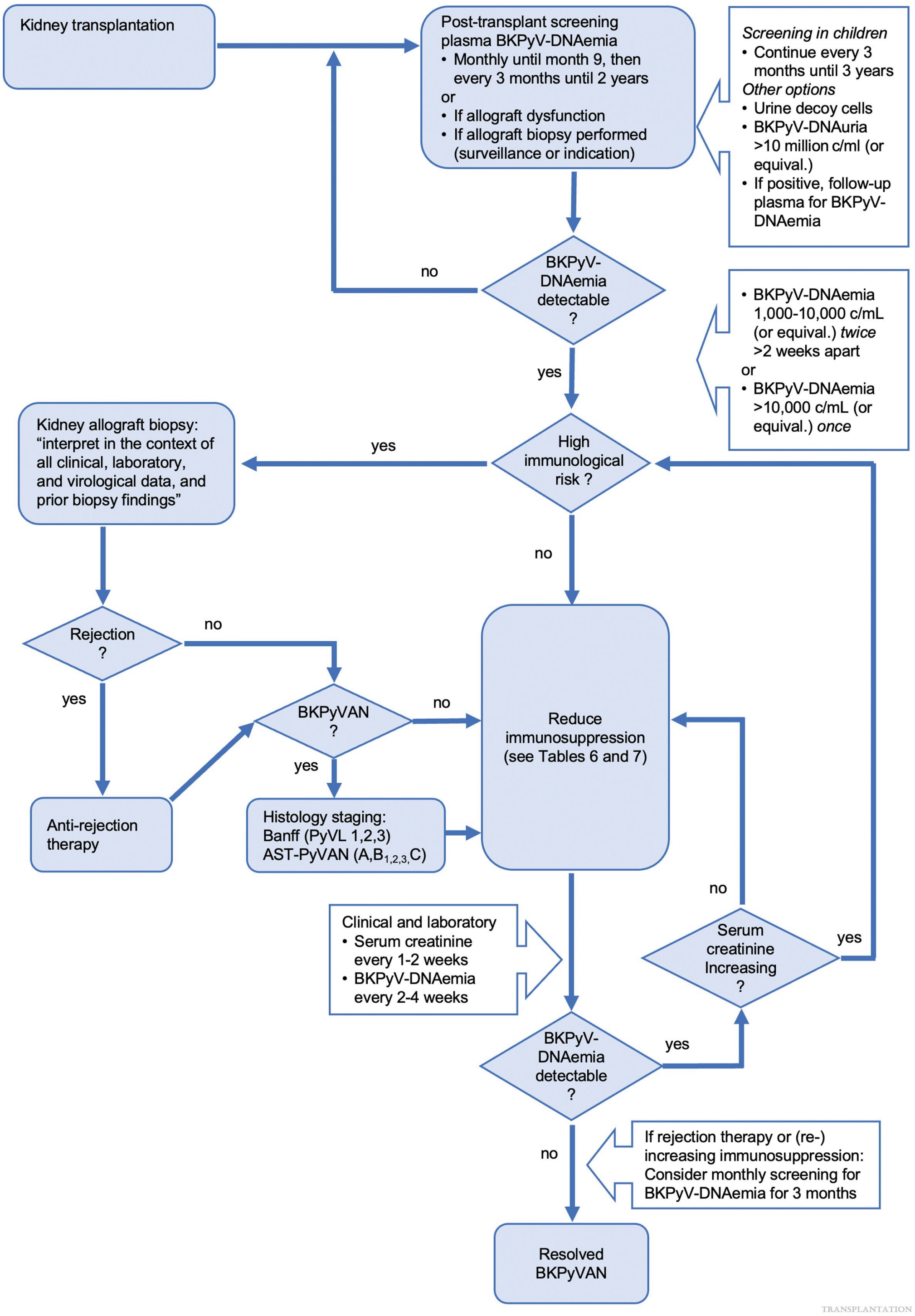

This figure was re-used from:

Kotton CN, Kamar N, Wojciechowski D, et al. on behalf of The Transplantation Society International BK Polyomavirus Consensus Group. The Second International Consensus Guidelines on the Management of BK Polyomavirus in Kidney Transplantation. Transplantation 108(9):p 1834-1866, September 2024. (DOI:10.1097/TP.0000000000004976), and accessed at: [https://journals.lww.com/transplantjournal/fulltext/2024/09000/the_second_international_consensus_guidelines_on.7.aspx].
This figure is published under a CC-BY-NC-ND license and attributed to the original authors.
